# m6A Modification Mediates Endothelial Cell Responses to Oxidative Stress in Vascular Aging Induced by Low Fluid Shear Stress

**DOI:** 10.1155/2023/8134027

**Published:** 2023-01-27

**Authors:** Zhijue Xu, Peng Qiu, Yihong Jiang, Jiateng Hu, Zhaoyu Wu, Jiahao Lei, Hongji Pu, Qun Huang, Xin Wang, Bo Li, Kaichuang Ye, Xinwu Lu, Guang Liu

**Affiliations:** ^1^Department of Vascular Surgery, Shanghai Ninth People's Hospital, Shanghai Jiao Tong University School of Medicine, Shanghai 200011, China; ^2^Vascular Center of Shanghai Jiao Tong University, Shanghai 200011, China; ^3^Department of Vascular Surgery, Fengcheng Hospital of Fengxian District, Shanghai 200011, China

## Abstract

N6-methyladenosine (m6A) is one of the most prevalent, abundant, and internal transcriptional modification and plays essential roles in diverse cellular and physiological processes. Low fluid shear stress (FSS) is a key pathological factor for many cardiovascular diseases, which directly forces on the endothelial cells of vessel walls. So far, the alterations and functions of m6A modifications in vascular endothelial cells at the low FSS are still unknown. Herein, we performed the transcriptome-wide m6A modification profiling of HUVECs at different FSS. We found that the m6A modifications were altered earlier and more sensitive than mRNA expressions in response to FSS. The low FSS increased the m6A modifications at CDS region but decreased the m6A modifications at 3′ UTR region and regulated both the mRNA expressions and m6A modifications of the m6A regulators, such as the RBM15 and EIF3A. Functional annotations enriched by the hypermethylated and hypomethylated genes at low FSS revealed that the m6A modifications were clustered in the aging-related signaling pathways of mTOR, PI3K-AKT, insulin, and ERRB and in the oxidative stress-related transcriptional factors, such as HIF1A, NFAT5, and NFE2L2. Our study provided a pilot view of m6A modifications in vascular endothelial cells at low FSS and revealed that the m6A modifications driven by low FSS mediated the cellular responses to oxidative stress and cell aging, which suggested that the m6A modifications could be the potential targets for inhibiting vascular aging at pathological low FSS.

## 1. Introduction

N6-methyladenosine (m6A) is one of the most prevalent, abundant, and internal transcriptional modification. More than 25% of human mRNAs are modified with m6A [1]. It is the methylation at the N6 position of adenosine in RNAs and has been discovered with diverse functions in cellular and physiological processes, and in many diseases such as cancer and obesity [2, 3]. The m6A is controlled by the m6A regulators including m6A methyltransferases (also called as the “writers”), demethylases (also called as the “erasers”), and m6A-binding proteins (also called as the “readers”) [4]. These regulators ensure m6A modifications as a dynamic and reversible process. In mRNAs, m6A was found enriched around 5′ untranslated terminal region (UTR), stop codon, and 3′ UTR [5]. Thus, m6A can regulate mRNA splicing, processing, translation, and stabilities [6, 7]. Recent studies revealed that m6A also plays essential roles in hypertension and cardiovascular diseases [8].

Shear force is particularly essential in the cardiovascular system [9]. It is the frictional force from blood flow and parallel to the vessel wall, therefore also called the fluid shear stress (FSS) [10]. Quantitatively, the FSS is defined as the force per unit area with the dyne/cm^2^ as unit [11]. Vascular endothelial cells are the direct objective cells of FSS in vessel walls [12]. Physiologically, persistent FSS maintains the viability of vascular cells and the integrity of vessel walls by inducing the release of vascular factors from endothelial cells [13, 14]. However, in many pathological conditions such as in the atherosclerosis, the major cause for coronary and peripheral artery diseases, FSS is decreased significantly [15]. Low FSS was reported to induce the inflammations, cell apoptosis, and oxidation stress, which could further remodel and aggravate the pathological changes of vessel walls [16, 17]. Thus, low FSS was considered as one of the key pathological factors for many cardiovascular diseases [18].

Aging is one of the major risk factors for the occurrence of cardiac-cerebral vascular diseases (CCVD) [19]. About 1/3 of all deaths at the age of 65 were caused by CCVD in the United States, while the data increased to 2/3 at the age of 85 [20]. Vascular aging is initialed with the endothelial dysfunction and the morphological changes of vessel walls [21]. Endothelial dysfunction is caused by many pathophysiological processes in endothelial cells, such as oxidative stress, epigenetic alterations, chronic inflammation, genomic instability stress, and disorder of proteomic homeostasis [22]. For example, the role of oxidative stress such as increasing of superoxide anion (O^2−^) in vascular disease is widely accepted [23]. Activation of Nrf2 (nuclear factor erythroid 2-related factor 2), a well-known inhibitor of oxidative stress, could inhibit the reactive oxygen species (ROS) generation and reduce the diabetes-induced vascular aging [24]. Low FSS is also associated with vascular aging and oxidative stress in endothelial cells. Recent studies reported that the FSS-driven sialic acid glycosylation regulated the Nrf2-mediated signaling and modulated oxidative stress in human endothelial cells [25].

So far, however, less studies have reported the relationship between m6A modifications and low FSS, and whether the alterations of m6A modifications driven by low FSS play roles in vascular aging is also unclear. Herein, to determine how low FSS regulates m6A modifications in vascular endothelial cells, we performed the transcriptome-wide m6A modification profiling of HUVECs at different FSS including high FSS, low FSS, and the stationary culture with no FSS. Comparative analysis and bioinformatics analysis were executed for the overall view of the functions of m6A at low FSS and discovering the relationship of m6A modifications with the cell aging and cellular response to oxidative stress at low FSS.

## 2. Materials and Methods

### 2.1. Cell Culture

The Human Umbilical Vein Endothelial Cells (HUVECs) were purchased from the Shanghai Cell Bank of the Chinese Academy of Sciences. HUVECs were cultured in High Glucose Dulbecco's Modified Eagle Medium, supplemented with 10% fetal bovine serum, 100 *μ*g/ml streptomycin, and 100 U/ml penicillin and incubated in a 5% CO_2_ atmosphere at 37°C.

### 2.2. Shear Stress

The fluid flow shear stress was performed on the Flexcell Flow System (Burlington, Ontario, Canada). In brief, the HUVECs were seeded on the CS-C culture Slips—collagen type I (Flexcell, Burlington, Ontario, Canada) for 12 h at 37°C. The fluid flow shear stress was applied using a Flexcell FX-4000 strain unit (Flexcell, Burlington, Ontario, Canada) with loading of 0, 5, and 15 dyne/cm^2^ for 12 h.

### 2.3. Phalloidin Staining

The HUVECs were treated with shear stress with loading of 0, 5, and 15 dyne/cm^2^ for 12 h, followed by fixing with 4% paraformaldehyde for 30 min. The fixed cells were permeabilized by methanol for 30 min, and then washed with PBS for 3 times. The cells were stained with 0.5 *μ*g/ml of iFluor™ 488-labeled phalloidin for 1 h, followed by washing with PBS for 3 times. The cells were further stained with 5 *μ*g/ml of DAPI for 30 min, followed by washing with PBS for 3 times. After that, the cells were imaged by Nikon A1Si laser-scanning confocal microscope (Tokyo, Japan).

### 2.4. RNA-Sequencing and m6A-Sequencing

The shear-stressed HUVECs were lysed via TRIzol regent (Thermo Fisher, USA) as soon as the shear stress finished. The total RNA was extracted by the RNeasy Mini Kit (QIAGEN, Germany) in accordance with the manufacturer's procedure. Chemically fragmented RNA (∼100 nucleotides) was incubated with m6A antibody for immunoprecipitation according to the standard protocol of the magna-methylated RNA immune-precipitation (MeRIP) m6A kit (Merck Millipore). Library preparation and high-throughput sequencing were performed by SHBIO (Shanghai, China). The transcriptome and purified RNA fragments from m6A-MeRIP were sequenced with Illumina HiSeq4000.

### 2.5. Data Analysis and Statistics

The quality visualization of all raw reads was accomplished by FastQC (version 0.11.9). Sequencing reads were aligned to the human genome GRCh38/hg38 using HISAT2 with default parameters. For the input libraries, the FPKMs were calculated and the differential gene expression analysis was performed using the R package DESeq2 (version 1.36.0). The |log2FC| > 1 and *P*-adjusted < 0.05 were used. For the m6A-modified peaks, the mapped reads were provided for the R package exomePeak (version 3.1) to identify the m6A peaks, followed by visualizing with IGV software. All called peaks were annotated by intersection with gene architecture using R package ChIPseeker (version 1.32.0). The peaks with fold enrichment of pooled to input >1 were considered as m6A-modified peaks. The fold changes of fold enrichments >2 or <0.5, and *P*-adjusted < 0.05 were used for differentially m6A-modified gene screening. Comparisons between two groups were performed using Student's *t* tests (two–tailed) or the Mann–Whitney *U* test.

### 2.6. Bioinformatics Analysis

The volcano plot was performed using GraphPad Prism 5.0 software (San Diego, CA, USA) with the fold changes and adjust *P* values. PCA analysis was conducted using the R package factoextra (version 1.0.7) and visualized using scatterplot3d (version 0.3-41). Venn diagram was conducted using the R package ggplot2 (version 3.3.3). The distribution of m6A modifications on genes was visualized via metageneplot. The motifs were analyzed using HOMER. Heatmaps were drawn using the R package ComplexHeatmap (version 2.12.0). The enrichment analysis of gene ontology (GO) and Kyoto Encyclopedia of Genes and Genomes (KEGG) was implemented by the R package clusterProfiler based on the package of http://org.Hs.eg.db and GOplot. The top five annotations of each database were shown in bar plot. The top 12 annotations with adjusted *P* values were shown in circle plot. The enrichment analysis of Reactome was conducted using the online database (https://reactome.org/). The top five annotations of hypermethylated genes and hypomethylated genes were shown in bar plot, respectively. Protein–protein interaction networks were constructed using STRING (version 11.5). The cutoff value of confidence interaction score was used >0.99. The networks were visualized with Cytoscape (version 3.8.2) and clustered with the Molecular Complex DEtection (MCODE) plugin. The total of 962 oxidative stress-related genes were obtained from the GeneCards® The Human Gene Database (downloaded on July 11, 2022), and the total of 1,509 human aging-related genes were obtained from the Human Aging Genomic Resources (downloaded on July 11, 2022).

## 3. Results

### 3.1. The m6A Modifications Were Regulated by Shear Stress

**Figure 1:**
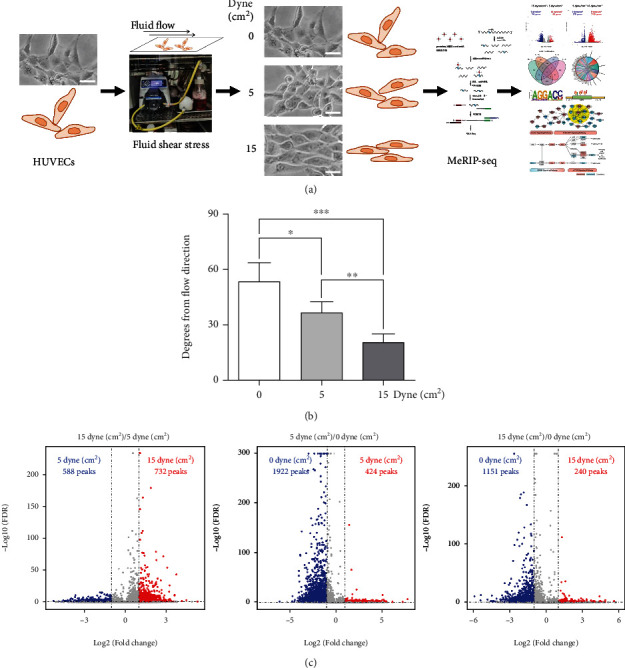
Fluid shear stress dramatically regulates the m6A modifications on mRNA. (a) Schematic of the MeRIP sequencing analysis of HUVECs induced by fluid shear stress. The HUVECs were stimulated by fluid shear stress for 12 h. (b) Quantitation of the angles between long cell axis and flow direction. (c) Volcano plots of the hypermethylated and hypomethylated peaks regulated by different fluid shear stress.

To determine the relationship between m6A modifications and shear stress, the HUVECs were treated with low FSS (5 dyne/cm^2^), high FSS (15 dyne/cm^2^), and no FSS (0 dyne/cm^2^), followed by MeRIP sequencing analysis (Figure 1(a)). Along with the dose of FSS from 0 to 15 dyne/cm^2^, cell alignments displayed more and more parallel (Figure 1(b)), confirming the cells at different FSS. After sequencing, compared to the no FSS, 891 genes were high-expressed at high FSS, and 2108 genes were high-expressed at low FSS (Figure S1). In the comparison of gene expressions at high and low FSS, 795 genes were upregulated at low FSS, while 196 genes were downregulated at low FSS (Figure S1). Moreover, 588 peaks were hypermethylated at low FSS and 732 peaks were hypomethylated at low FSS (Figure 1(c)). While by comparing to no FSS, less peaks were hypermethylated at both high and low FSS (Figure 1(c)).

**Figure 2:**
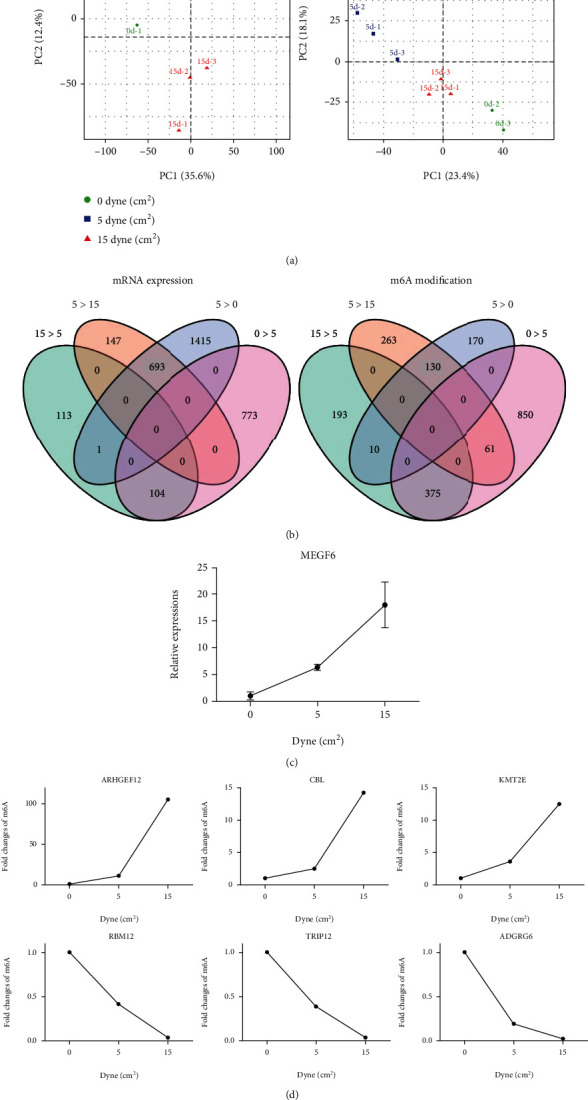
The m6A modifications displayed a better dose response to fluid shear stress. (a) PCA plots of the repeated cells exposed to different fluid shear stress. (b) Venn diagrams between differential expressed genes and hypermethylated/hypomethylated genes, respectively. (c) The gene expression of MEGF6 corresponding to different fluid shear stress. (d) Fold changes of m6A modifications on the mRNA of ARHEF12, CBL, KMT2E, RBM12, TRIP12, and ADGRG6, which were corresponding to different fluid shear stress.

Both gene expressions and m6A modifications revealed that the three groups of cells treated by no, low, and high FSS were separated significantly (Figures 2(a) and S2). Interestingly, the high FSS-treated cells fall in between no FSS and low FSS-treated cells (Figure 2(a)), suggesting that the differences between low FSS and no FSS were larger than that between high FSS and no FSS. We next used Venn diagram to analyze the overlapping among differently expressed or m6A-modified genes between high and low FSS and low and no FSS (Figure 2(b)). There was only one gene, MEGF6, displaying a significantly increasing expression from 0 to 15 dyne/cm^2^ shear stress (left panel of Figure 2(b), and Figure 2(c)). While there were 10 genes with increasing m6A modifications and 61 genes with decreasing m6A modification corresponding to the changes of 0 to 15 dyne/cm^2^ shear stress (right panel of Figure 2(b)). The top three of the increasing and decreasing m6A-modified genes were ARHGEF12, CBL, and KMT2E and RBM12, TRIP12, and ADGRG6, respectively (Figure 2(d)). This result indicated that in addition to the gene expressions, the m6A modifications displayed a better dose response to FSS. The gene expression of MEGF6 and those m6A modifications on the identified genes might be considered as biosensors of HUVECs in response to FSS.

### 3.2. Low FSS Altered the Distribution of m6A Peaks

**Figure 3:**
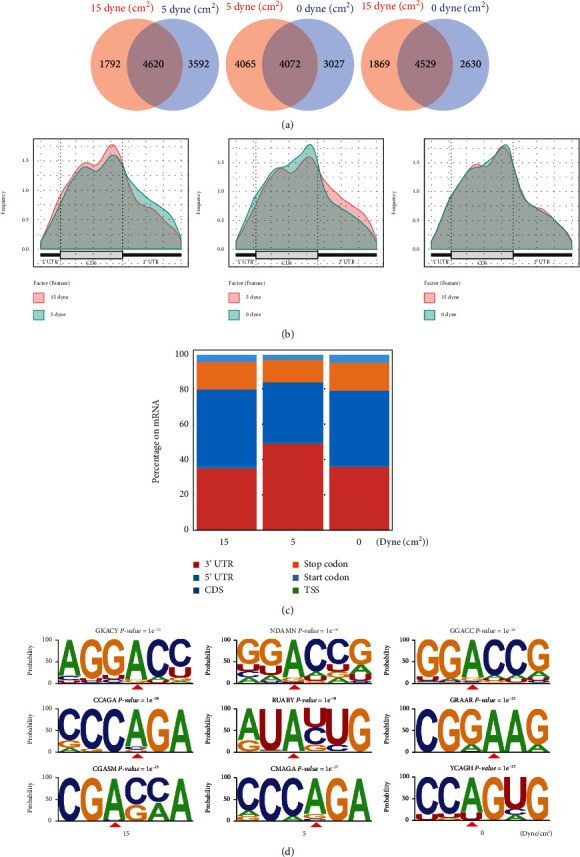
Low FSS altered the distribution of m6A peaks. (a) Venn diagrams of m6A modified peaks between no, low, and high FSS-treated cells. (b) Metageneplot of the m6A-modified peaks on mRNA. (c) Distribution of the m6A peaks to the respective input read coverage for mRNA. (d) Motifs at nucleotide positions around the m6A peaks. The top three motifs were displayed. The modified positions of adenine were labeled with red triangles.

Venn diagrams revealed that the overlapping m6A-modified peaks between low FSS and high FSS, low FSS and no FSS, and high FSS and no FSS were 4620, 4072, and 4529, respectively (Figure 3(a)). Using these overlapping peaks, we analyzed the distribution of m6A modifications stimulated by low and high FSS. Compared to no FSS, low FSS reduced the m6A modifications in CDS region of mRNAs and increased the m6A modifications in 3′ UTR regions. While high FSS altered a fat lot of m6A distributions to no FSS (Figure 3(b)). Moreover, compared to high FSS, low FSS reduced the m6A modifications in CDS region from 43.3% to 33.4% and increased the m6A modifications in 3′ UTR region from 35.8% to 49.6% (Figures 3(b) and 3(c)), indicating that the low FSS could enhanced the m6A deposition on 3′ UTR region. To validate the m6A sites, we performed de novo motif search of the m6A peaks with HOMER and identified the first motif of all three groups were GGACC (Figure 3(d)), which was consistent with the m6A consensus DRACH motif. Compared to high FSS, low FSS showed the motif with enrichment of uracil (Figure 3(d)), further suggesting that low FSS altered the distribution and sequence preference of m6A modifications.

### 3.3. Low FSS Modulates the m6A Regulators

**Figure 4:**
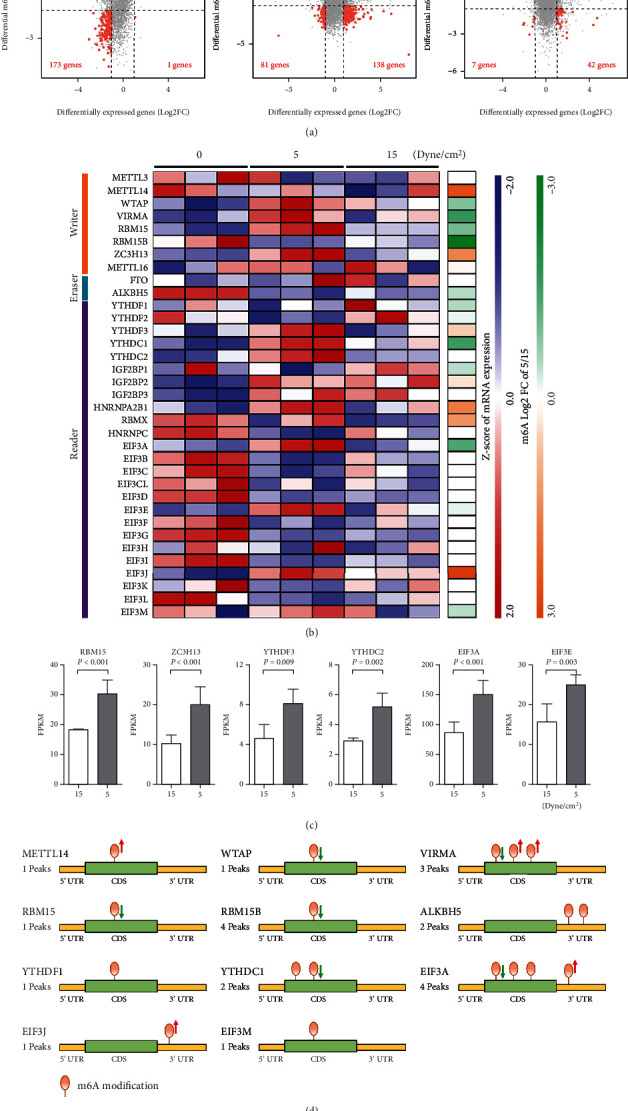
Low FSS modulates the m6A regulators. (a) Four-quadrant diagram of the differentially expressed genes and differentially m6A-modified genes. (b) Heatmap of the m6A regulators in mRNA expressions and m6A modifications. (c) RNA expressions of six differentially expressed m6A regulators. |FoldChange| > 1.5, *P*-adjusted < 0.05. (d) Distributions and counts of m6A modifications on differentially modified m6A regulators. *P*-adjusted < 0.05. The hypermethylated and hypomethylated peaks were labeled with red and green arrows, respectively.

To determine the correlation between mRNA expressions and m6A modifications under FSS treatments, we performed the four-quadrant diagrams as shown in Figure 4(a). In comparation between low and high FSS, only 7 genes of high FSS group were high-expressed and differentially modified by m6A with 6 of them hypermethylated and one of them hypomethylated. But in low FSS group, 215 genes were high-expressed and differentially modified by m6A with 42 genes hypermethylated and 173 genes hypomethylated. It indicated that low FSS dramatically altered m6A modification in HUVECs.

To explore the mechanism of low FSS in regulation on m6A, we analyzed the expressions and m6A modifications of the m6A regulators including 8 writers, two erasers, and 25 readers (Figure 4(b)). Among the 35 m6A regulators, 6 of them were differential expressed (Figure 4(c)), and 11 of them were differentially modified by m6A (Figure 4(d)). The 6 differential expressed regulators were all upexpressed in low FSS, in which the writer of RBM15 was hypomethylated and the reader of EIF3A was four-sites of methylation with one hypomethylated and one hypermethylated. These results indicated that low FSS altered the m6A modification and expression of the m6A regulators especially RBM15 and EIF3A, which further regulated the m6A modifications on the other mRNAs.

### 3.4. Low FSS Modulated the m6A Modifications of the Aging-Related Genes

**Figure 5:**
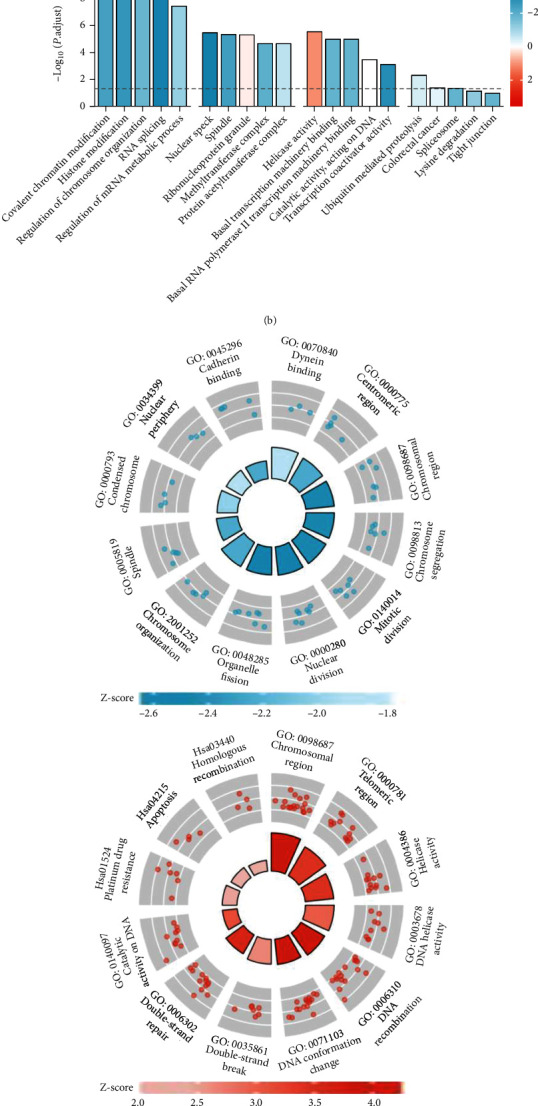
Low FSS modulated the m6A modifications on aging-related genes. (a) Scatter plot of the annotation enrichment analysis (GO and KEGG databases) based on fold changes in the m6A modification (*x*-axis) and transcriptome (*y*-axis). The annotations upregulated in m6A-modified genes were colored in red, and annotations downregulated in m6A-modified genes were colored in blue. (b) Bar plot of the annotation enrichment analysis (GO and KEGG databases) of m6A-modified genes. (c) Circle plot of GO analysis of m6A-modified genes. The hypermethylated and hypomethylated genes were analyzed separately. (d) Interaction network of m6A-modified genes, analyzed by STRING and visualized with Cytoscape software. The hypermethylated and hypomethylated genes were colored in red and blue, respectively. The top five functional clusters were defined *via* the MCODE plugin.

To determine the biofunctions of the m6A-modified genes regulated by low FSS, we comparatively analyzed the enriched annotations of the m6A modified genes and the differentially expressed genes in according to GO and KEGG databases (Figure 5(a)). There were 110 annotations enriched in both two gene sets. Among them, 32 annotations displayed the consistent changes with 31 of them upregulated in both two gene sets, and one of them was downregulated which was the GO annotation of establishment or maintenance of cell polarity. On the other hand, 78 of annotations were upregulated in mRNA expression set but were downregulated in m6A modification set. It indicated that the m6A modifications modulated by low FSS were dramatically upregulated many processes in HUVECs, no matter hypermethylation nor hypomethylation. We next focused on the annotations enriched by m6A-modified gene set. In the integrated analysis of both hypermethylated and hypomethylated gene sets, most of the enriched annotations were with negative z-scores (Figure 5(b)). It suggested that the low FSS might play more functions via hypomethylation rather than hypermethylation. GO analysis revealed that low FSS-hypomethylated genes were enriched in the biological processes of RNA splicing, histone modification, and covalent chromatin modification (Figure 5(c)). The proteins translated by those genes were located in nuclear speck, spindle, spliceosomal complex, methyltransferase complex, and pronucleus and played molecular functions of thyroid hormone receptor binding, basal transcription machinery binding, basal RNA polymerase II transcription machinery binding, and transcription coactivator activity (Figure 5(c)). It indicated that low FSS played roles in gene transcriptions and RNA processing, which were all important in cell proliferation and survival. We further performed the interaction network of the m6A-modified genes using the STRING database. The low FSS-driven hypermethylated and hypomethylated genes displayed strong interactions, and the network under the confidence interaction score of 0.99 as cutoff value was shown (Figure 5(d)). The top five of MCODE clusters were lined with the score of 6.86, 5.60, 5.60, 4.50, and 4.0, respectively. The first clusters contained the genes of JUN, HIF1A, SP1, MYC, SMAD3, EP300, KAT2B, and CREBBP, which were transcriptional factors or cofactors and all played roles in aging-related processes such as cell cycles, stress response, and senescence. Taken together, these results all indicated that the low FSS-driven m6A modifications were enriched in the aging-related genes.

### 3.5. The Differential m6A Modifications Regulated Cell Response to Oxidative Stress

**Figure 6:**
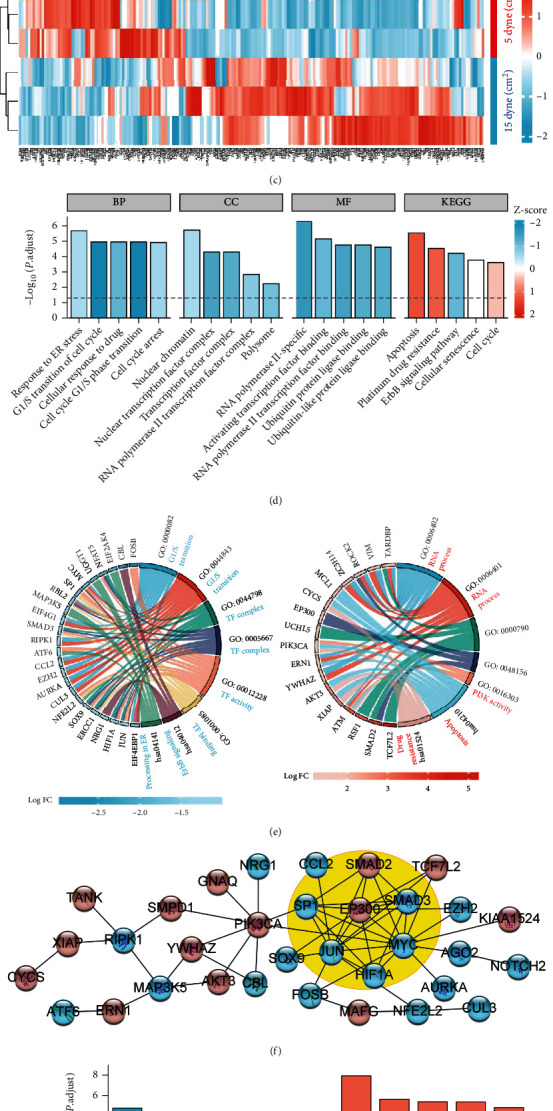
Essential roles of the m6A modifications at low FSS in response to oxidative stress. (a) Venn diagram of m6A-modified genes and oxidative stress-related genes. (b) Volcano plots of the m6A-modified oxidative stress-related genes. (c) Heatmap of the m6A-modified oxidative stress-related genes. (d) Bar plot of GO and KEGG database analyses. (e) Circle plot of GO and KEGG database analyses. The hypermethylated and hypomethylated genes were analyzed separately. The annotations of hypermethylated genes were colored in red, and annotations of hypomethylated genes were colored in blue. (f) Interaction network of differentially m6A-modified oxidative stress-related genes. The hypermethylated and hypomethylated genes were colored in red and blue, respectively. The first functional clusters were circled out in yellow. (g) Bar plot of Reactome database analysis. The annotations of hypermethylated genes were colored in red, and annotations of hypomethylated genes were colored in blue.

To determine the functional role of the m6A modifications in response to oxidative stress at low FSS, we compared the literature oxidative stress-related genes with our detected m6A-modified genes (Figure 6(a)). Among the 962 oxidative stress-related genes, 210 of them were modified with m6A with 30 hypermethylated genes and 41 hypomethylated genes at low FSS (Figures 6(b) and 6(c)). Functional enrichment analysis indicated that most of the enriched annotations were with negative z-score (Figure 6(d)), suggesting that the functions of hypomethylated genes at low FSS were more assembled than that of the hypermethylated genes. The hypomethylated genes were enriched in G1/S transition, TF complex, TF activity, TF binding, ErbB signaling, and processing in ER, and the hypermethylated genes were enriched in the RNA process, PI3K activity, apoptosis, and drug resistance (Figure 6(e)). These annotations were important for aging-related processes, such as cell cycle, protein synthesis, and cell survival. Moreover, the protein-protein interaction network revealed that the first MCODE cluster with a score of 6.67 contained the transcriptional factors of JUN, SP1, HIF1A, MYC, EP300, SMAD2, and SMAD3 (Figure 6(f)), which were also important for cell cycle and survival. In the Reactome database analysis, the hypomethylated genes were enriched in intrinsic pathway for apoptosis, apoptosis, and transcriptional regulation by TP53, SMAC (DIABLO) binds to IAPs, and FOXO-mediated transcription, and the hypermethylated genes were enriched in the signaling by NOTCH, Pre-NOTCH transcription and translation, cellular responses to stimuli, estrogen-dependent gene expression, and cellular responses to stress (Figure 6(g)). Taken together, these results confirmed that the m6A modifications on the oxidative stress-related genes driven by low FSS could play essential roles in aging process.

### 3.6. The m6A Modifications Regulated Aging Process at Low FSS

**Figure 7:**
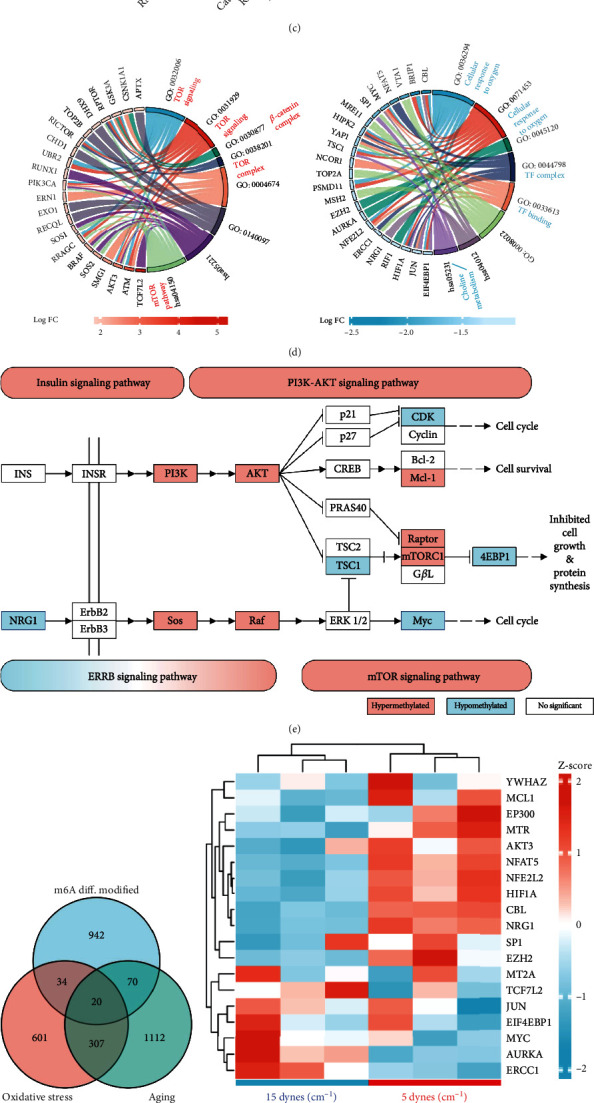
The low FSS-driven m6A modifications regulated aging process. (a) Venn diagram of m6A-modified genes and aging-related genes. (b) Volcano plots of the m6A-modified aging-related genes. (c) Bar plot of GO and KEGG database analyses. (d) Circle plot of GO and KEGG database analyses. The hypermethylated and hypomethylated genes were analyzed separately. (e) KEGG pathway enriched by m6A-modified aging-related genes. The full maps of the four KEGG pathways were shown in Figure S4 and S5. (f) Venn diagram of m6A differentially modified genes, oxidative stress-related genes, and aging-related genes. (g) Heatmap of the m6A differentially modified genes which was both oxidative stress and aging-related genes. (h) RNA expressions of the genes in Figure 7(g) with adjust *P* value < 0.05.

To determine how the m6A modifications regulates the aging process at low FSS, we performed the functional enrichment analysis of the overlapping genes of aging-related genes with our detected m6A-modified genes. There were 1,509 aging-related genes in the Human Aging Genomic Resources. Among them, 384 of them were modified with m6A (Figure 7(a)), with 47 hypermethylated genes and 65 hypomethylated genes at low FSS (Figure 7(b) and Figure S3). GO analysis indicated that the hypomethylated genes were enriched in the cellular response to oxygen, TF complex, and choline metabolism (Figures 7(c) and 7(d)), which were assistant with the annotations in the response to oxidative stress (Figure 6(e)). And the hypermethylated genes were enriched in mTOR signaling, *β*-catenin complex, and TOR complex (Figures 7(c) and 7(d)). KEGG pathway analysis further indicated that the m6A at low FSS was upregulated in mTOR signaling pathway, insulin signaling pathway, and PI3K-AKT signaling pathway and were abnormal in ERRB signaling pathway (Figure 7(e)). In detail, the PI3K, AKT, Mcl-1, Sos, Raf, Raptor, and mTORC1 were hypermethylated, and the NRG1, TSC1, Myc, 4EBP1, and CDK were hypomethylated. These pathways played essential roles in cell cycle, cell survival, inhibition of cell growth, and protein synthesis, which were the core pathways of cell aging. Thus, the low FSS might regulate the aging process via altering the m6A of mTOR, PI3K-AKT, insulin, and ERRB signaling pathways.

We further comprehensively analyzed the oxidative stress-related genes, aging-related genes, and the low FSS-driven m6A differentially modified genes. There were 20 of m6A differentially modified genes which were related with both the response to oxidative stress and the aging (Figure 7(f)), including many important transcriptional factors such as HIF1A, SP1, JUN, MYC, and NFAT5 (Figure 7(g)). Nine of the 20 genes were differentially expressed at mRNA level with *P* value < 0.05 (Figure 7(h)), with seven upexpressed genes and two downexpressed genes. Eight of them were hypomethylated and one was hypermethylated. The transcriptional factors of NFAT5, HIF1A, and NFE2L2 were all hypomethylated and upexpressed (Figure 7(h)). Taken together, these results indicated that the low FSS might regulate the aging process via hypomethylating the key transcriptional factors and signaling pathway in the response to oxidative stress.

## 4. Discussion

Low FSS and m6A modifications were both widely studied in the aging process, but the internal relationship between the two is unknown so far. In this study, we performed the transcriptome-wide m6A modification profiling of HUVECs at different FSS and compared the alteration of m6A at the physiological high FSS and the pathological low FSS. Functional annotations were enriched more in the gene set with hypomethylation at low FSS, which were clustered in the processes of cell cycle, apoptosis, and cellular senescence, and in the genes of transcriptional factors and RNA processing. Our results revealed that the m6A modifications driven by low FSS could mediate the cellular responses to oxidative stress and cell aging.

Changes in m6A modifications have been found with close interactions and relationships with oxidative stress in many physiological and pathological processes [26]. In human keratinocyte cells, high level of m6A modification in human HaCaT cells promoted cell survivals through inhibiting oxidative stress [27]. In the development of Parkinson's disease, inhibition of m6A modification was found to elevate oxidative stress and Ca^2+^ influx that resulted in the cell death of dopaminergic neuron [28]. We found that low FSS-modulated oxidative stress-related processes including hypermethylated RNA process, cell apoptosis, and drug resistant genes and hypomethylated cell cycle, protein process, and transcriptional factor genes. Noteworthy, a group of oxidative stress-related transcriptional factors were highly enriched, including HIF1A, MYC, and SP1. The HIF1A (hypoxia inducible factor 1*α*) is a critical factor in oxidative stress, which is induced by hypoxia and many other oxidative stress inducers, and regulated cellular responses to modulate oxidative stress [29]. A recent study reported that hypermethylation of HIF1A mRNA facilitated the mRNA translation and increased HIF1A protein abundance [30]. In our results, low FSS hypomethylated the HIF1A mRNA. It indicated that the protein abundance of HIF1A in endothelial cells might be inhibited at low FSS, suggesting that low FSS could induce oxidative stress. The MYC is also essential for the cellular response to oxidative stress [31]. Increasing global m6A RNA modification in leukemia cells could decrease the stability of MYC and result in the suppression of MYC pathways [32]. At low FSS, the global of m6A was preferred to hypomethylation, which might active the MYC pathways in response to oxidative stress, also suggesting that low FSS induced oxidative stress. Furthermore, the SP1 was found inducible by oxidative stress for negative feedback of antideath role [33]. And inhibition of m6A on SP1 was found to decrease SP1 expression and inhibit cell proliferation [34]. Our results indicated that low FSS also hypomethylated SP1, suggesting that low FSS inhibit the antioxidative stress functions of SP1. Taken together, the hypomethylations of all the three transcriptional factors of HIF1A, MYC, and SP1 revealed a consistent cellular fate that low FSS promoted oxidative stress in vascular endothelial cells. Therefore, increasing the m6A modifications might be a potential target for modulating the low FSS-driven oxidative stress in vessels.

On the other hand, oxidative stress can also mediate the change of m6A modification via regulating the expression of m6A regulators. In cobalt-induced oxidative stress, the expressions of the m6A demethylase FTO were inhibited, which further regulated caspase activation, G1/S cell cycle arrest, and cell apoptosis in an m6A-dependent manner [35]. In placenta trophoblast, another m6A demethylase of ALKBH5 was found upexpressed in preeclamptic placental tissues and mediated the m6A modification of peroxisome proliferator-activated receptor PPARG, which alleviates hypoxia-induced oxidative stress and apoptosis [36]. In our case of low FSS, the expressions of FTO and ALKBH5 were found with no significant changes, while the methyltransferase of RBM15 and the m6A binding proteins of EIF3A were upexpressed and m6A differentially modified. Both RBM15 and EIF3A were crucial in cell cycle and cell survival [37, 38]. RBM15-mediated m6A was found playing roles in the insulin signaling pathway [39], which was also enriched in our results at low FSS. EIF3A was found to directly bind with 5′ UTR of ATF4, a critical antagonizing factor of oxidative stress in cardiovascular disease [40], and regulated the protein expression of ATF4 in a m6A dependent-manner [41]. Thus, low FSS might use RBM15 and EIF3A as handles to modulate m6A modifications in vascular endothelial cells and to mediate oxidative stress.

Low FSS is a profound pathological inducer for endothelial dysfunction and vascular aging [42]. We identified that the low FSS-driven m6A modifications were closely associated with aging-related processes such as cell cycle and several signaling pathways including mTOR signaling pathway, PI3K-AKT signaling pathway, insulin signaling pathway, and ERBB signaling pathway. Low FSS mediated the arrest of cell cycle in both endothelial cell and tumor cells via the functions of p21 and SMAD family [43, 44]. The phosphorylation of SAMD2/3 was recently reported being activated at low FSS to mediate artery remodeling [45]. We found that the m6A of SMAD2/3 mRNAs were also differentially modified at low FSS, suggesting that low FSS regulated the SMAD signaling at not only posttranslational modification level but also posttranscriptional modification level. m6A was found to modulate mTOR and PI3K-AKT signaling pathway in many cancer cells, and the hypermethylation-mediated via METTL14 deactivated the PI3K-AKT and mTOR signaling and suppressed gastric cancer cell proliferation and aggression [46]. In our vascular endothelial cells, the mTOR and PI3K-AKT signaling genes were hypermethylated at low FSS, suggesting that it might inhibit the pathway activations and the cell proliferation as well as the roles in cancer cells. Therefore, the low FSS-driven m6A modifications might induce the cell cycle arrest and proliferation suppression. Together with the roles of m6A in oxidative stress, low FSS might promote endothelial cell aging via mediating abnormal modifications of m6A.

Mechanosensors for FSS such as protein of cytoskeleton, proteoglycan of heparan sulfate, and transmembrane of Ca^2+^ ions have been widely studied in last decades [47, 48]. Dynamic modifications, not only posttranslational modification but also posttranscriptional modification, were changed more immediately in cellular stress responses than their protein and mRNA backbones [49]. In our study, at the mRNA level and m6A modification level, we found that only one gene, MEGF6, shown a consistent trend of mRNA expression to the changes of FSS, while the m6A of 71 genes has the consistent trends to the changes of FSS. It suggested the m6A modifications were more suitable as biosensors of FSS than mRNA expressions. The changes of m6A modifications might be one of the initiated steps for vascular endothelial cells in response to abnormal FSS.

Aging is one of the key risk factors for cardiovascular disease [42]. As a pathological inducer for aging, low FSS mediated the endothelial dysfunctions and the development of almost all kinds of cardiovascular diseases such as atherosclerosis and abdominal aortic aneurysm [50, 51]. The relationship of low FSS with many conventional pathways has been intensively studied, while that with the posttranslational or transcriptional modifications were investigated just at the beginning step. Dynamic modifications provide the functional diversities for the modified backbones and play fine-tuning roles in many cellular processes [52]. Our study provided a proof-of-concept that low FSS mediated the dynamic changes of m6A in vascular endothelial cells which might participate in the regulating of oxidative stress and aging. Further experimental evidences were needed in future studies.

## 5. Conclusion

In summary, we performed the transcriptome-wide m6A modification profiling of HUVECs at different FSS. In comparison of the mRNA expressions, m6A modifications displayed a better linear relationship with the increasing of FSS, indicating that the m6A modifications were altered earlier and more sensitive than mRNA expressions in response to FSS. Compared to the physiological high FSS of 15 dyne/cm^2^, the pathological low FSS of 5 dyne/cm^2^ increased the m6A modifications at CDS region but decreased the m6A modifications at 3′ UTR region. Among the m6A regulators, the writer of RBM15 and the reader of EIF3A were both differentially expressed and differentially m6A-modified at low FSS. Functional enrichment analysis revealed that the annotations were enriched more by the hypomethylated genes at low FSS. In cellular response to oxidative stress, the m6A modifications were clustered at the cell cycle, apoptosis, and cellular senescence, which were aging-related processes. Further analysis confirmed that low FSS might regulate the aging process via altering the m6A modification of mTOR, PI3K-AKT, insulin, and ERRB signaling pathways and via hypomethylating the key transcriptional factors in the response to oxidative stress, such as HIF1A, NFAT5, and NFE2L2. Our study provided a pilot view of the dynamic changes of m6A modifications in vascular endothelial cells at low FSS and revealed that the m6A modifications driven by low FSS mediated the cellular responses to oxidative stress and cell aging, which also suggested that m6A modifications could be the potential targets for regulating vascular aging at pathological low FSS.

## Data Availability

The datasets are available from the corresponding authors upon reasonable request.

## Abbreviations

CDS: Coding sequence
FSS: Flow shear stress
GO: Gene ontology
HUVECs: Human umbilical vein endothelial cells
KEGG: Kyoto encyclopedia of genes and genomes
m6A: N6-methyladenosine
MCODE: Molecular complex detection
MeRIP: Methylated RNA immunoprecipitation
mTOR: Mammalian target of rapamycin
ROS: Reactive oxygen species
UTR: Untranslated region.

## Supplementary Materials

Supplementary Materials Supplementary File. Figure S1: volcano plots of the differential expressed genes regulated by different flow shear stress. Figure S2: 3D and 2D PCA plots of the repeated cells exposed to different flow shear stress. (a) The gene mRNA expression level. (b) The m6A modification level. Figure S3: heatmap of the m6A-modified aging-related genes. Figure S4: the intact KEGG map of the insulin signaling pathway (up panel) and the PI3K-AKT signaling pathway (bottom panel). The ugregulated genes were colored in red, and the downregulated genes were colored in green. Figure S5: The intact KEGG map of the ERBB signaling pathway (up panel) and the mTOR signaling pathway (bottom panel). The ugregulated genes were colored in red, and the downregulated genes were colored in green. (Supplementary Materials).
