# Supplementary material for: m6A Modification Mediates Endothelial Cell Responses to Oxidative Stress in Vascular Aging Induced by Low Fluid Shear Stress
Source: Oxid Med Cell Longev. 2023 Jan 27;2023:8134027. doi: 10.1155/2023/8134027 (PMC9897929; doi:10.1155/2023/8134027)
Supplement: Supplementary Materials — Supplementary File. Figure S1: volcano plots of the differential expressed genes regulated by different flow shear stress. Figure S2: 3D and 2D PCA plots of the repeated cells exposed to different flow shear stress. (a) The gene mRNA expression level. (b) The m6A modification level. Figure S3: heatmap of the m6A-modified aging-related genes. Figure S4: the intact KEGG map of the insulin signaling pathway (up panel) and the PI3K-AKT signaling pathway (bottom panel). The ugregulated genes were colored in red, and the downregulated genes were colored in green. Figure S5: The intact KEGG map of the ERBB signaling pathway (up panel) and the mTOR signaling pathway (bottom panel). The ugregulated genes were colored in red, and the downregulated genes were colored in green. (Supplementary Materials). [file 8134027.f1.docx]

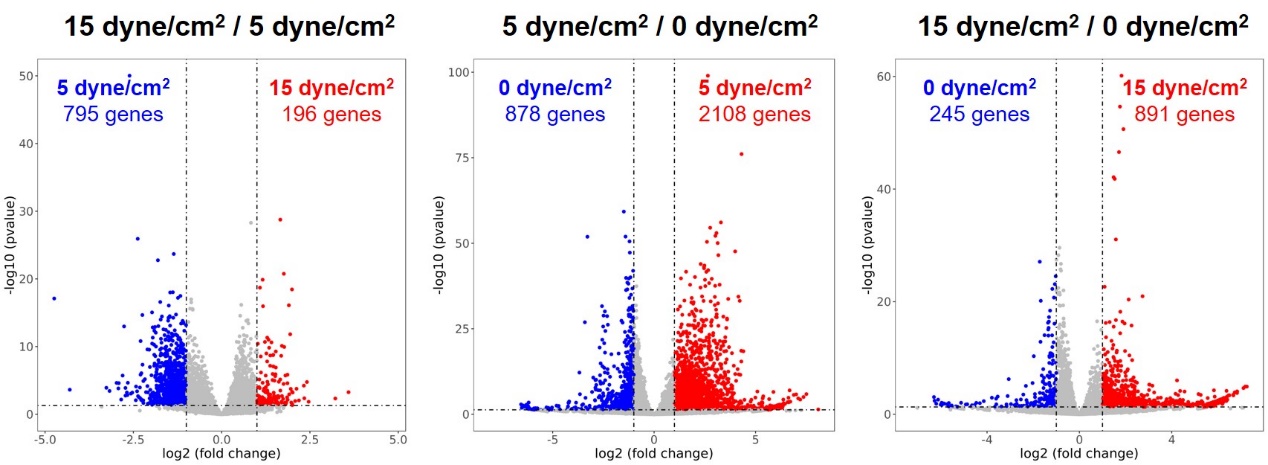


Figure S1. Volcano plots of the differential expressed genes regulated by different flow shear stress.


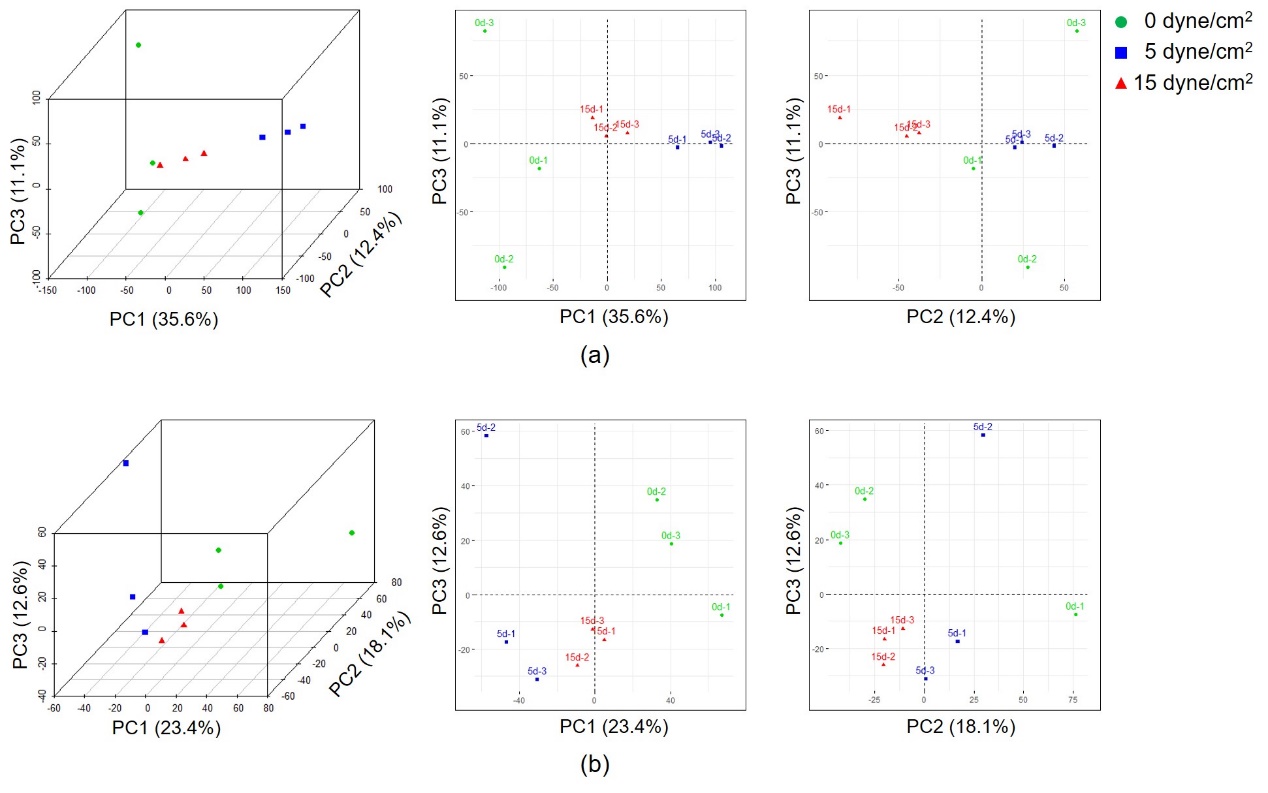


Figure S2. 3D and 2D PCA plots of the repeated cells exposed to different flow shear stress. (a) the gene mRNA expression level. (b) the m6A modifications level.


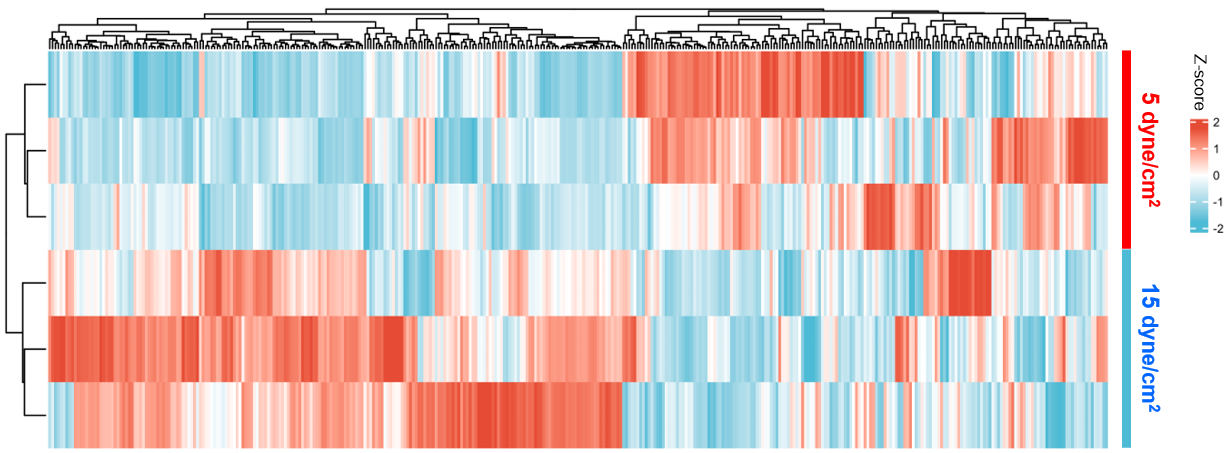


Figure S3. Heatmap of the m6A modified aging related genes.


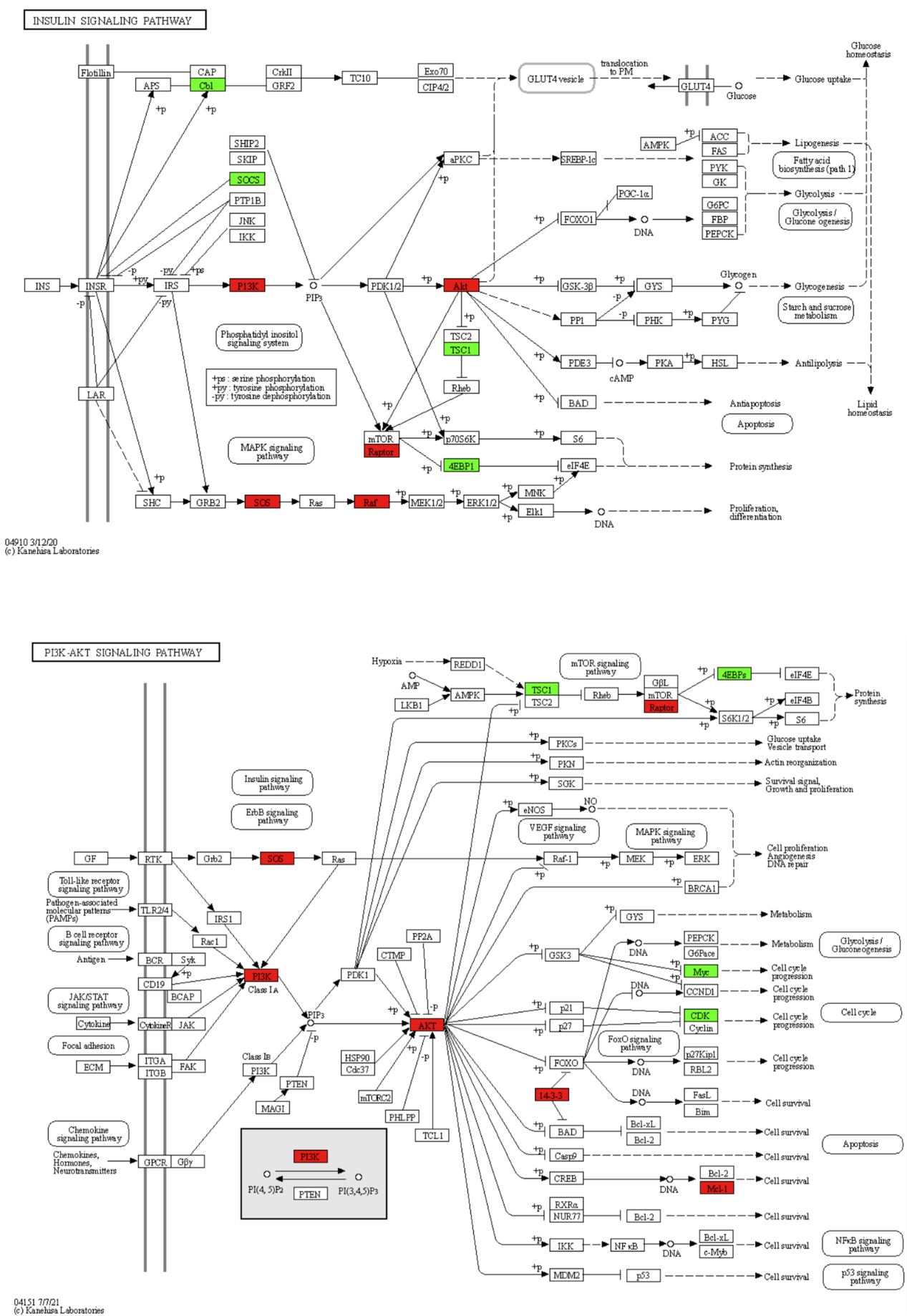


Figure S4. The intact KEGG map of the insulin signaling pathway (up panel) and the PI3K-AKT signaling pathway (bottom panel). The ug-regulated genes were colored in red, and the down-regulated genes were colored in green.


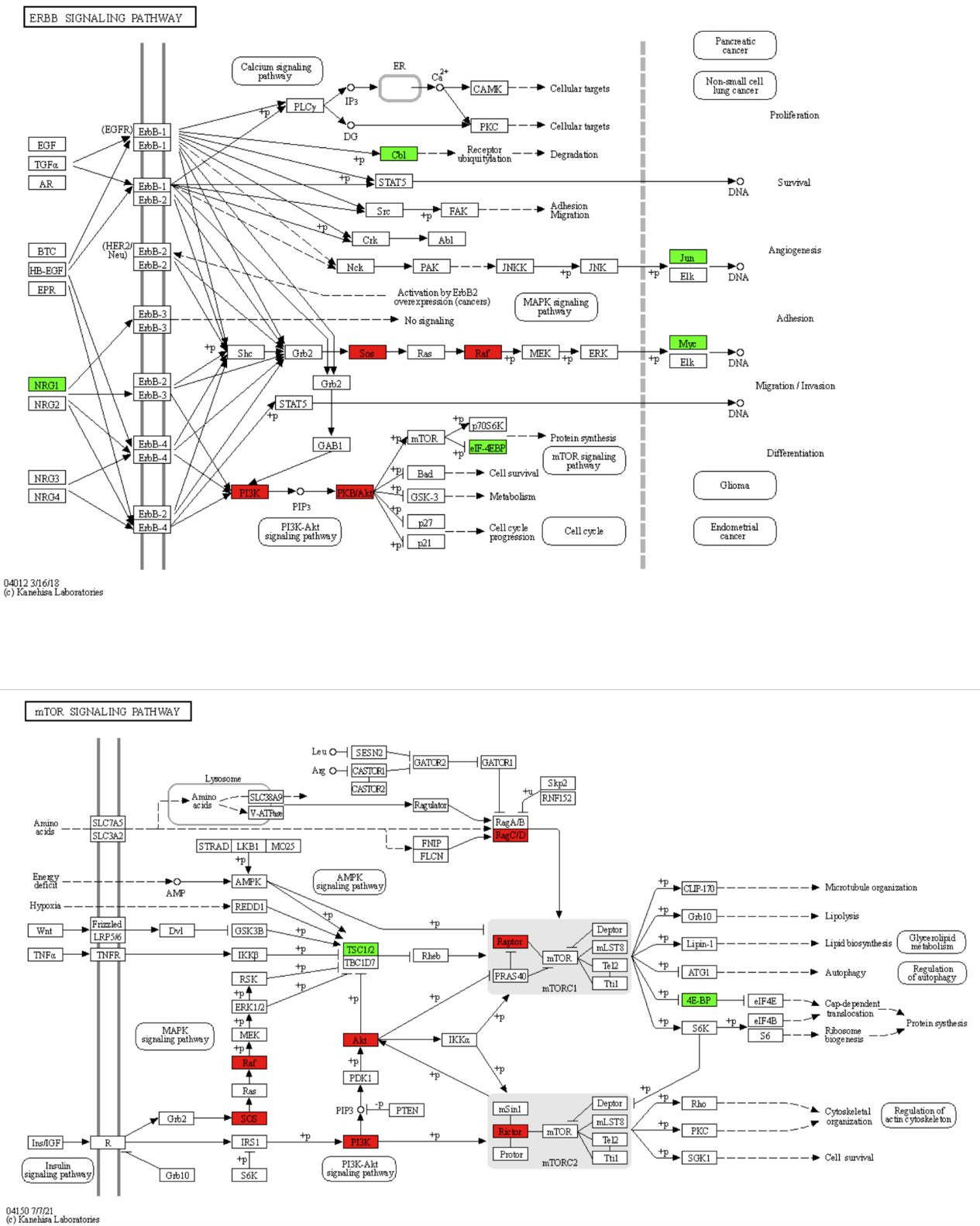


Figure S5. The intact KEGG map of the ERBB signaling pathway (up panel) and the mTOR signaling pathway (bottom panel). The ug-regulated genes were colored in red, and the down-regulated genes were colored in green.
